# IL-17A Enhances Microglial Response to OGD by Regulating p53 and PI3K/Akt Pathways with Involvement of ROS/HMGB1

**DOI:** 10.3389/fnmol.2017.00271

**Published:** 2017-08-31

**Authors:** Bin Zhang, Ning Yang, Zhi-Ming Mo, Shao-Peng Lin, Feng Zhang

**Affiliations:** ^1^Department of Neurology, the Fifth Affiliated Hospital of Guangzhou Medical University Guangzhou, China; ^2^Department of Emergency, the Second Affiliated Hospital of Guangzhou Medical University Guangzhou, China; ^3^Department of Neurosurgery, the Fourth Affiliated Hospital of Guangzhou Medical University Guangzhou, China

**Keywords:** IL-17A, reactive oxygen species, HMGB1, microglial cells, cerebral ischemia-reperfusion injury, proteomics, mass spectrometry

## Abstract

Cerebral ischemia-reperfusion injury (IRI) has a complex pathogenesis, and interleukin-17 (IL-17) is a newly identified class of the cytokine family that plays an important role in ischemic inflammation. An oxygen-glucose deprivation (OGD) model showed that IL-17A expression was significantly up-regulated in microglial cells. After IL-17A siRNA transfection, the inhibition of proliferation, and the increased apoptosis in microglial cells, induced by OGD/reperfusion, was improved, and the elevation of Caspase-3, Caspase-8, Caspase-9, and poly ADP ribose polymerase (PARP) activities was inhibited. Mass spectrometry demonstrated that IL-17A functioned through a series of factors associated with oxidative stress and apoptosis and regulated Caspase-3 activity and apoptosis in microglial cells via the p53 and PI3K/Akt signaling pathways. IL-17A, HMGB1, and ROS were regulated mutually to exhibit a synergistic effect in the OGD model of microglial cells, but the down-regulation of IL-17A or HMGB1 expression did not completely inhibit the production of ROS. These findings demonstrated that ROS might be located upstream of IL-17A and HMGB1 so that ROS can regulate HMGB1/IL-17A expression to affect the p53 and PI3K/Akt signaling pathways and therefore promote the occurrence of apoptosis in microglial cells. These findings provide a novel evidence for the role of IL-17A in ischemic cerebral diseases.

## Introduction

Cerebral ischemia is a common disease that severely endangers human health, and it is characterized by a high incidence, high disability rate, high mortality, and high recurrence rate (Mellado et al., 2005; Chen et al., 2013). Thus, approaches to alleviate cerebral ischemia-reperfusion injury (IRI), increase the success rate of rescue and surgery for patients with cerebral ischemia and improve the patients' quality of life are urgently needed.

Many pathogenic mechanisms are involved in the development and progression of cerebral IRI, such as the excessive release of excitatory amino acids, a dysregulation of inflammatory reaction, oxidative stress, and apoptosis (Ten and Starkov, 2012; Mozaffarian et al., 2015). Recently, local excessive inflammation following cerebral ischemia-reperfusion (IR) has been shown to be a major factor causing reperfusion injury, and the inflammatory response is reported to inhibit neuronal regeneration and other alterations associated with the functional recovery from stroke (Lakhan et al., 2009). Interleukin-17 (IL-17) is a key cytokine that plays an important role in ischemic inflammation. A remarkable increase in IL-17, IL-1β, and IL-8 expression is detected in the peripheral blood mononuclear cells of most patients with cerebral ischemia (Kostulas et al., 1999). By *in situ* hybridization and immunohistochemistry, IL-17 expression gradually increases in the cerebrum tissues of rats from 1 h to 6 days following cerebral ischemia, and the T cell infiltration correlates positively with the area of the cerebral injury, which demonstrates that IL-17 and T cells are involved in cerebral injury (Li et al., 2005). In addition, the T cells, IL-23, and IL-17 that infiltrate into the ischemic cerebral tissues is critical to cerebral infarction formation and neurologic impairment, and IL-17 is reported to play an important role in the delayed phase of ischemia and reperfusion, namely, at the time of neuronal cell apoptosis and necrosis (Shichita et al., 2009). In a model of cerebral IRI, a significant reduction is observed in the loss of neurological function, the area of the infarction and the mortality of IL-17^−/−^ and IL-23p19^−/−^ mice (Witowski et al., 2004). It is therefore considered that IL-17 is of great significance in the development and progression of cerebral IRI.

As a pro-inflammatory factor, IL-17 induces the up-regulation of chemotactic factor expression and the infiltration of inflammatory cells in tissues (Witowski et al., 2004; Weaver et al., 2006). However, IL-17 expression shows an increasing tendency in a mouse model of middle cerebral artery occlusion (MCAO) starting at day 2 after the cerebral ischemia and reaches a peak on day 6. During this period, the blood flow is interrupted in the local cerebral tissues, and the T cells cannot infiltrate, which is when inflammation may be mainly mediated by neuroglial cells (Li et al., 2001; Maślińska et al., 2002). Under co-stimulation of IL-1β and IL-23, microglial cells express a large amount of IL-17 (Kawanokuchi et al., 2008). In addition, the toll-like receptor-2 (TLR-2)/IL-23/IL-17 signaling pathway induces IL-17 release from the microglial cells, resulting in neuronal cell damage during the period of reperfusion (Lv et al., 2011). Therefore, IL-17, produced by microglial cells, plays an important role in the late phase of brain injury. As an inherent immune effector cell in the brain, microglial cells exhibit an immunosurveillance role, and produce a cascade reaction to the injury of central neurological system (Ling et al., 2001; Kaur et al., 2007). The results of these studies further demonstrate that microglial cells are primary cells that mediate post-ischemic inflammation (Soltys et al., 2005).

In addition, IL-17, produced by microglial cells, is of great significance in cerebral IRI. However, the exact mechanism has not been demonstrated until now. In this study, the protein expression profile regulated by IL-17A was isolated and identified in the oxygen-glucose deprivation (OGD) model of microglial cells using two dimensional (2D) gel electrophoresis and matrix-assisted laser desorption/ionization tandem time-of-flight mass spectrometry (MALDI-TOF/TOF-MS) technology, and the signaling pathway in which IL-17 functioned was validated *in vitro*, which provides new insights into therapy of cerebral ischemia.

## Materials and methods

### Cell culture

Microglia were isolated from primary mixed glial cell cultures prepared from newborn C57BL/6J mice (Guangdong Medical Lab Animal Center, China) on day 10 by shaking the flasks overnight at 300 rpm on a rotary shaker at 37°C. Purified microglia were resuspended and cultured in complete medium containing 1% microglia growth supplement (ScienCell, USA) for 2–3 days. The purity of the cultures was almost 100%, as determined by immunostaining with an anti-Iba1 antibody (Abcam, USA). The protocols for the animal experiments were approved by the Animal Experiment Committee of Guangzhou Medical University. Mouse EOC 2 microglial cells were obtained from the American Type Culture Collection (http://www.ATCC.org) and were cultured in high-glucose Dulbecco's Modified Eagle Medium (DMEM; Gibco-BRL) containing 10% fetal bovine serum (FBS; Gibco-BRL).

### Cell transfection

The cells were seeded in 6-well plates at 1 × 10^5^ cells/ml/well 1 day before the transfection. The following day the transfection was performed when the cells reached ~70% confluence. The final concentration of IL-17A or HMGB1 siRNA (Santa Cruz) was 100 nM. The transfection was conducted with the X-tremeGENE siRNA Transfection Reagent (Roche) according to the manufacturer's instructions. The transfection medium was replaced 4–6 h after the transfection. After siRNA transfection, Tenovin-6 (10 μM; SantaCruz, USA) and MK-2206 (5 μM; Selleck, China) were added or not.

### Oxygen-glucose deprivation reperfusion treatment and ELISA

After transfection for 24 h, the cells were replaced medium with glucose-free Earle's balanced salt solution, and then placed in an oxygen deprived incubator (95% N_2_/3% CO_2_/2% O_2_) at 37°C for 2 h. The control cells were incubated in Earle's balanced salt solution with 10 mM glucose under normal conditions (95% air/5% CO_2_) for the same period of time. The cells were moved to normal conditions to terminate the OGD and start reperfusion. The protein concentrations of IL-17A (eBioscience, USA) and HMGB1 (SHINO-TEST, Japan) in the culture supernatants were determined by an ELISA kit following the manufacturer's protocol.

### MTT assay

The growth-inhibitory effect was measured using the standard 3-(4,5-dimethylthiazol-2-yl)-2,5-diphenyltetra-zolium bromide (MTT) assay. Microglial cells (6 × 10^4^ cells/well) were seeded in a 96-well tissue culture microplate for 24 h, and then, the cells were exposed to the different treatments. After that, 25 μl of the MTT solution (5 mg/ml in phosphate buffered saline) was added into each well and was incubated for 4 h at 37°C. The metabolized MTT product was dissolved in DMSO and was quantified by measuring the optical density at 570 nm on a microplate reader (Dynex Technologies, USA).

### Evaluation of cell proliferation

Cell proliferation was estimated in 96-well plates using a colorimetric immunoassay, based on the measurement of BrdU incorporation during DNA synthesis (BrdU ELISA kit, Roche Diagnostics, Germany). The cells were labeled with BrdU (10 mM 5-bromo-2′-deoxyuridine) for 3 h at 37°C. Cells were fixed, and incubated with peroxidase-conjugated anti-BrdU antibody for 90 min at room temperature. Then the peroxidase substrate 3,3′,5,5′-tetramethylbenzidine was added, and BrdU incorporation was quantitated by differences in absorbance at wavelength 370 minus 492 nm. Cell proliferation was expressed as the mean percentage of the control values (set at 100%).

### Flow cytometric analysis

There are certain characteristics of apoptotic cells that can be identified and used to detect apoptotic cells in an otherwise healthy population of cells. Technically the easiest characteristic to detect is loss of DNA from permeabilized cells due to DNA fragmentation. When cells are permeabilized, for example by 70% ethanol, the fragmented 182 bp DNA multimers leak out of the cell. The result is a population of cells with a reduced DNA content. If the cells are then stained with a DNA intercalating dye like propidium iodide, then a DNA profile representing cells in G1, S-phase, and G2M will be observed with apoptotic cells being represented by a sub G0/G1 population seen to the left of the G0/G1 peak. Microglial cells were washed with PBS and fixed in 75% ethanol overnight at –20°C. The fixed cells were stained with PI (1.21 mg/ml Tris, 700 U/ml RNase, 50.1 μg/ml PI, pH 8.0) for 4 h in the dark. The red PI fluorescence was measured with a Coulter Epics XL flow cytometer (Coulter, Germany) using CXP software. In each sample, 10,000 events were measure for the apoptosis detection and cell cycle analysis. The data were analyzed with the Expo32 analysis tool. A DNA histogram represented the proportion of cells in the G0/G1, S, and G2/M phases. The apoptotic cells with a hypodiploid DNA content were measured by quantifying the sub-G1 peak in the cell cycle pattern.

### Assessment of the caspase and poly ADP ribose polymerase (PARP) activities

The activities of caspase-3, -8, -9, and PARP in the lysates of the microglial cells were monitored by a fluorometric method. The cell lysates and specific substrates were placed in 96-well plates and were then incubated at 37°C for 2 h. The enzymatic activities were determined by measuring the fluorescence intensity in a fluorescence microplate reader (MD) with the excitation and emission wavelengths at 380 and 440 nm, respectively.

### MALDI-TOF mass spectrometry and bioinformatics analysis

For the protein extraction, 2D-gel electrophoresis, image analysis, protein identification, tryptic digestion, and MALDI-TOF mass spectrometry, please see the Supplementary Methods (Data Sheet 1). The differentially expressed proteins identified by 2D-gel electrophoresis and MALDI-TOF/TOF-MS were aggregated and analyzed using the UNIPROT database and the DAVID data analysis software and were classified into GO (gene ontology) categories (http://www.geneontology.org/) according to their biological functions and the physiological processes in the body.

### Western blot analysis

The cells were collected, washed twice with PBS, and lysed in RIPA buffer containing protease inhibitors. After determining the protein concentration with the BCA Protein Assay Reagent Kit (Pierce), equal amounts of protein were separated on 8% SDS-PAGE, electrically transferred to a PVDF membrane and were blocked with 5% skim milk. The membranes were incubated with IL-17A (1:800; CST, USA), HMGB1 (1:800; CST, USA), Cleaved Caspase-3 (1:800; CST, USA), Osteopontin (1:600; Abcam, USA), p53/p-p53 (1:1,000; CST, USA), Akt/p-Akt (1:1,000; CST, USA), and β-actin (1:2,000; Sigma, USA) antibodies respectively, overnight at 4°C. After washing, the membranes were then incubated with a horseradish peroxidase (HRP)-conjugated anti-rabbit secondary antibody (Southern Biotech) at room temperature for 1 h. The membranes were finally incubated with a West Femto chemiluminescence substrate (Pierce), and the images were visualized and recorded.

### Measurement of ROS generation

The intracellular reactive oxygen species (ROS) levels were determined by a fluorometric assay (DCF-DA assay). Briefly, the microglial cells were seeded in 96-well microplates at 6 × 10^4^ cells/well for 24 h, and then, the cells were incubated with the different treatments for different periods of time. After incubation, the treated cells were incubated with 10 μM DCFH-DA at 37°C for 15 min. The medium was aspirated, and the cells were washed with PBS twice. Before the measurement, 100 μl of PBS was added into each well and the ROS was immediately measured. The generation of ROS was determined by the fluorescence intensity with the excitation and emission wavelengths set at 485 and 525 nm, respectively. The change in the intracellular ROS levels of each group was determined by calculating the ΔF = (F − F0)/F0, where F represents the fluorescence read at each time point, and F0 is the control fluorescence.

### TUNEL-DAPI co-staining assay

TUNEL-DAPI co-staining assay was used to analyze the apoptotic cells based on the *In situ* cell Death Detection kit (Roche). Briefly, 4% formaldehyde was used for fixation at 4°C for 25 min, and then, the cells was washed by PBS. Next, 0.2% Triton X-100 was then added into cells for a 5 min incubation. Then, the cells were mixed with 100 μl of Equilibration Buffer at room temperature for 10 min. The cells were washed with Saline Sodium Citrate (SSC) for 15 min after an incubation with 50 μl of the TUNEL reaction mixture, containing the nucleotide mixture and terminal deoxynucleotidyl transferase (TdT), for 60 min at 37°C. Moreover, the cells were incubated with 0.3% H_2_O_2_ for 10 min and a Streptavidin working solution for 30 min at room temperature. The cells were incubated with 0.5 μg/ml of DAPI in a humidified chamber in the dark for 5 min at room temperature. Eventually, a fluorescence microscope (Nikon Eclipse 80i) was used to examine and take photos of the stained cells.

### Statistical analysis

The experiments were carried out at least in triplicate, and the results were expressed as the mean ± *SD*. All variances are homogeneous and samples follow a normal distribution, so the differences between the two groups were analyzed by a two-tailed Student's *t*-test, while those between three or more groups were analyzed by a one-way analysis of variance (ANOVA). Differences with *P* < 0.05 (*) were considered statistically significant.

## Results

### IL-17A up-expression in an OGD model of microglial cells

**Figure 1:**
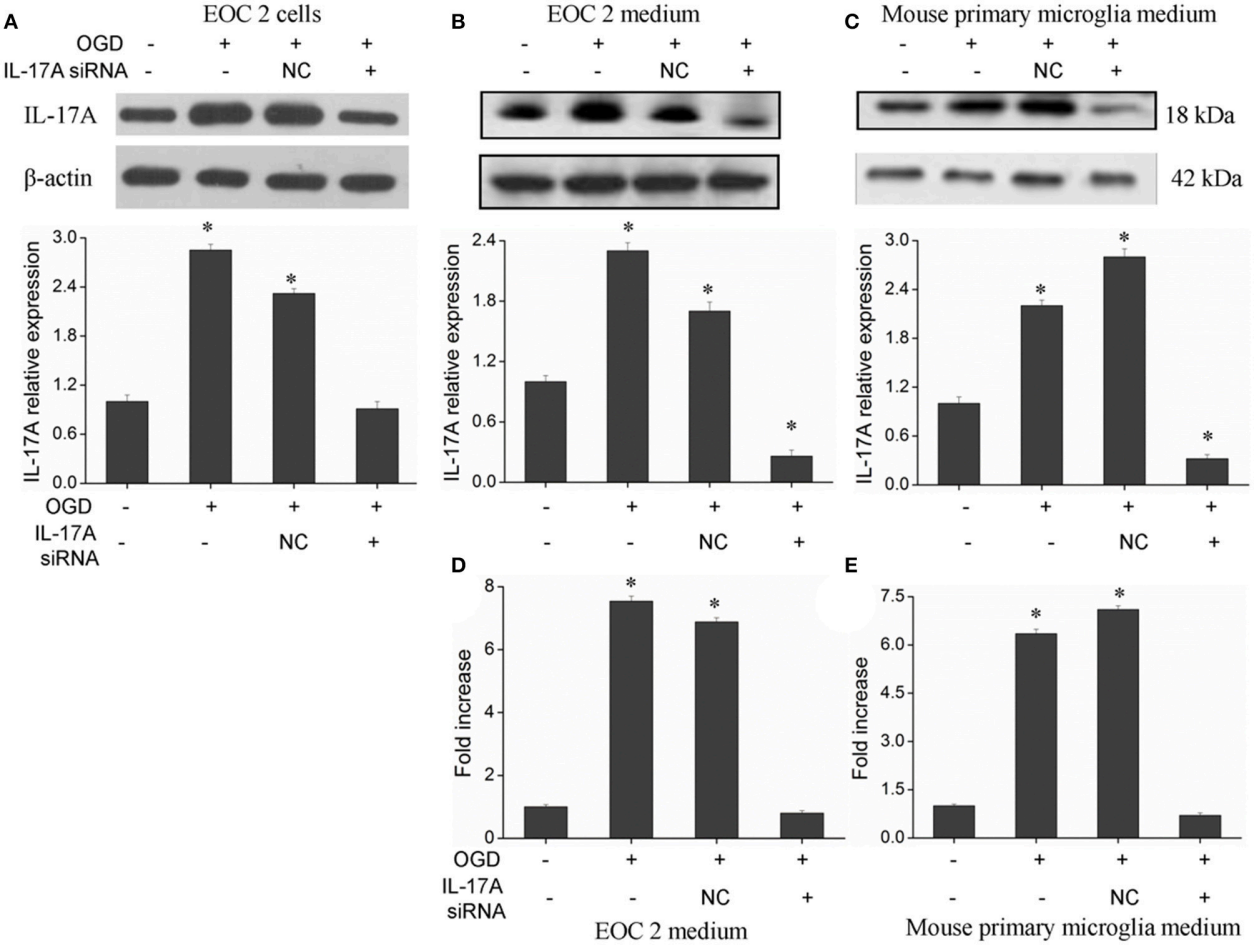
Expression of IL-17A in microglial cells. After transfection with IL-17A siRNA for 24 h, microglial cells were placed in an oxygen deprived incubator at 37°C for 2 h, and then moved to normal conditions to terminate the OGD and start reperfusion for 24 h. IL-17A expression as measured by Western blot analysis in EOC 2 cells **(A)**, EOC 2 culture supernatants **(B)**, and mouse primary microglia culture supernatants **(C)**. Protein concentrations of IL-17A in EOC 2 **(D)** and mouse primary microglia **(E)** culture supernatants were determined by ELISA. Experiments were carried out at least in triplicate and the results were expressed as the mean values, *P < 0.05 vs. the control. NC, negative control siRNA.

The IL-17 family includes six homologous members, including IL-17A, IL-17B, IL-17C, IL-17D, IL-17E, and IL-17F (Wang et al., 2009; Zhu and Qian, 2012), in which IL-17A and IL-17F receive more attention. IL-17A plays a more important role in autoimmune diseases than IL-17F (Ishigame et al., 2009), and is a cytokine with a strong pro-inflammatory action. In addition, IL-17A is a major factor that causes ischemia-reperfusion tissue injury (Li et al., 2005; Xue et al., 2011), and it causes inflammatory injury to local tissues and apoptosis through multiple pathways. However, the underlying mechanisms remain unclear. Therefore, the current study mainly focused on IL-17A. A model of OGD was established to simulate cerebral ischemia-reperfusion *in vitro*, and a significant up-regulation of IL-17A expression was found during OGD/reperfusion, while the addition of siRNA caused a reduction in IL-17A expression by using the expression of beta-actin in cells as internal reference (Figure 1). Using ELISA to detect IL-17A expression in the culture supernatants of EOC 2 and mouse primary microglia, the results were similar to those of the western blot (Figures 1D,E). Therefore, we concluded that the up-expression of IL-17A might play a critical role in microglial cells.

### Down-regulation of IL-17A promotes proliferation and inhibits apoptosis in microglial cells in an OGD model

**Figure 2:**
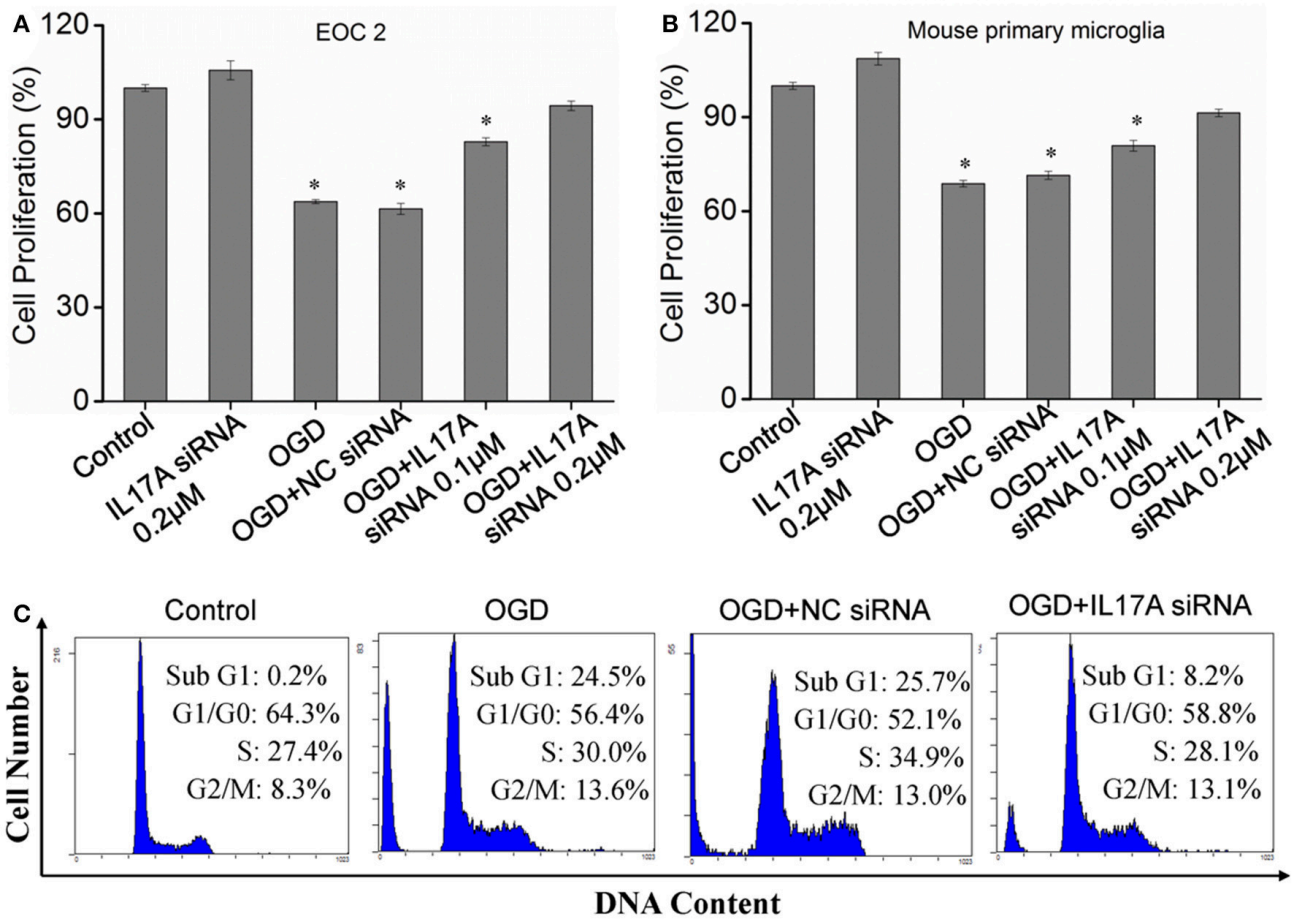
Effect of IL-17A siRNA on microglial cell proliferation and apoptosis in OGD model. The cells were transfected with IL-17A siRNA for 24 h, started the OGD for 2 h and reperfusion for 24 h. EOC 2 cells **(A)** and mouse primary microglia **(B)** proliferation was examined by MTT assay. *P < 0.05 vs. the control. **(C)** EOC 2 cells apoptosis as examined by flow cytometric analysis.

**Figure 3:**
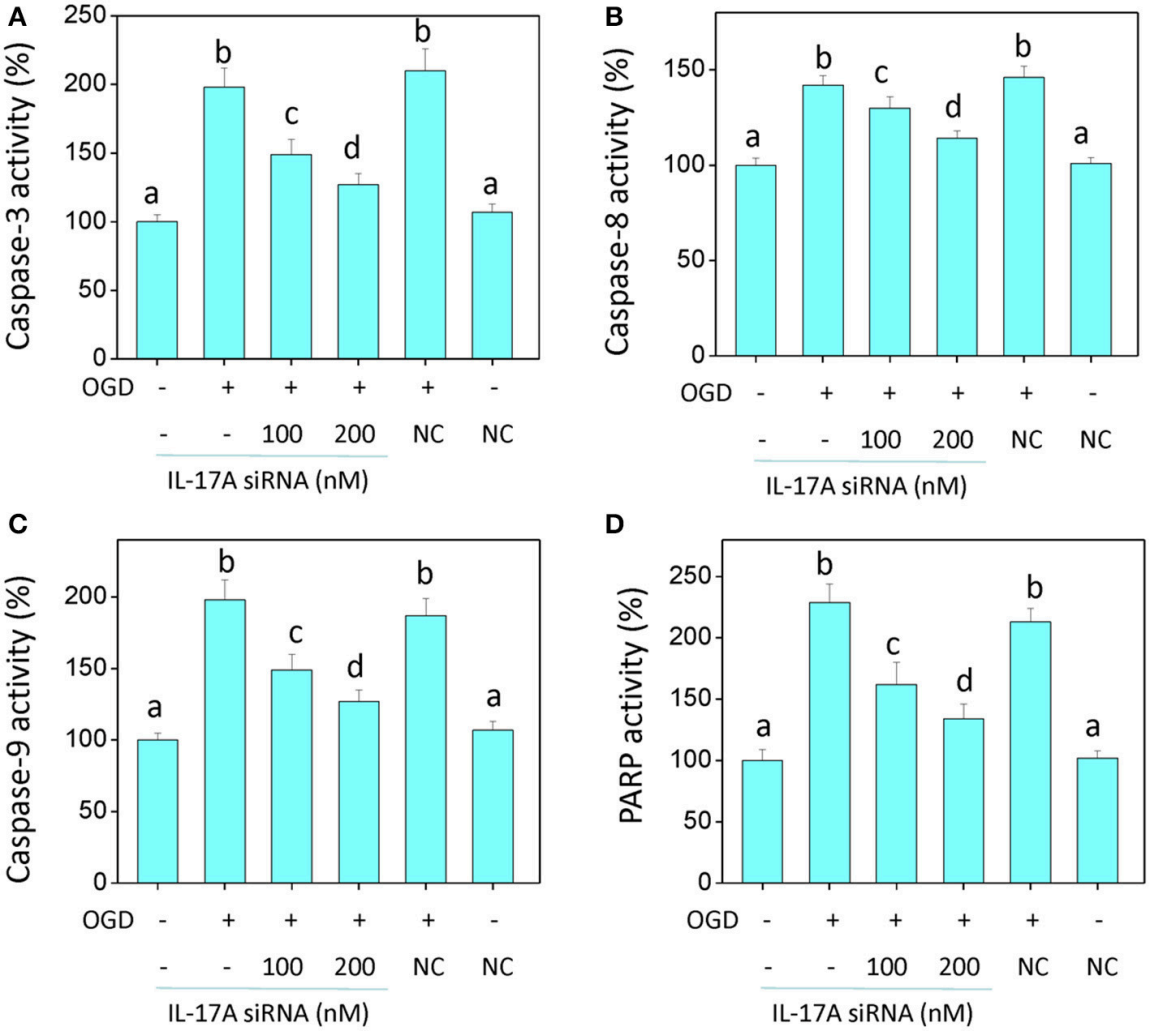
IL-17A siRNA suppresses OGD/reperfusion-induced caspase activation **(A–C)** and PARP cleavage **(D)** in microglial cells. EOC 2 cells were transfected with IL-17A siRNA for 24 h, then started the OGD for 2 h and reperfusion for 24 h. The activities of caspase-3, -8, -9, and PARP in the lysates of microglial cells were monitored by fluorometric method. Data are presented as the means ± *SD* from three independent experiments. Bars with different characters are significantly different at *P* < 0.05 level.

In the model of OGD, a decline in the proliferation and a significant elevation in apoptosis was observed in the microglial cells. When IL-17A expression was down-regulated, the number of proliferative microglial cells significantly increased, and the number of apoptotic cells reduced remarkably (Figure 2). The detection of apoptosis-related parameters showed that the activity of Caspase-3, Caspase-8, Caspase-9, and poly ADP ribose polymerase (PARP) was remarkably elevated in the microglial cells in the OGD model, while the down-regulation of IL-17A expression inhibited the activity of Caspase-3, Caspase-8, Caspase-9, and PARP, and the alteration of Caspase-3, Caspase-9, and PARP was most notable (Figure 3). Caspase-3, Caspase-9, and PARP are important factors associated with apoptosis, and IL-17A may exhibit a pro-apoptotic action via Caspase-3, Caspase-9, and PARP. Therefore, IL-17A is considered to play a critical role in microglial cells.

### Mass spectrometry

**Figure 4:**
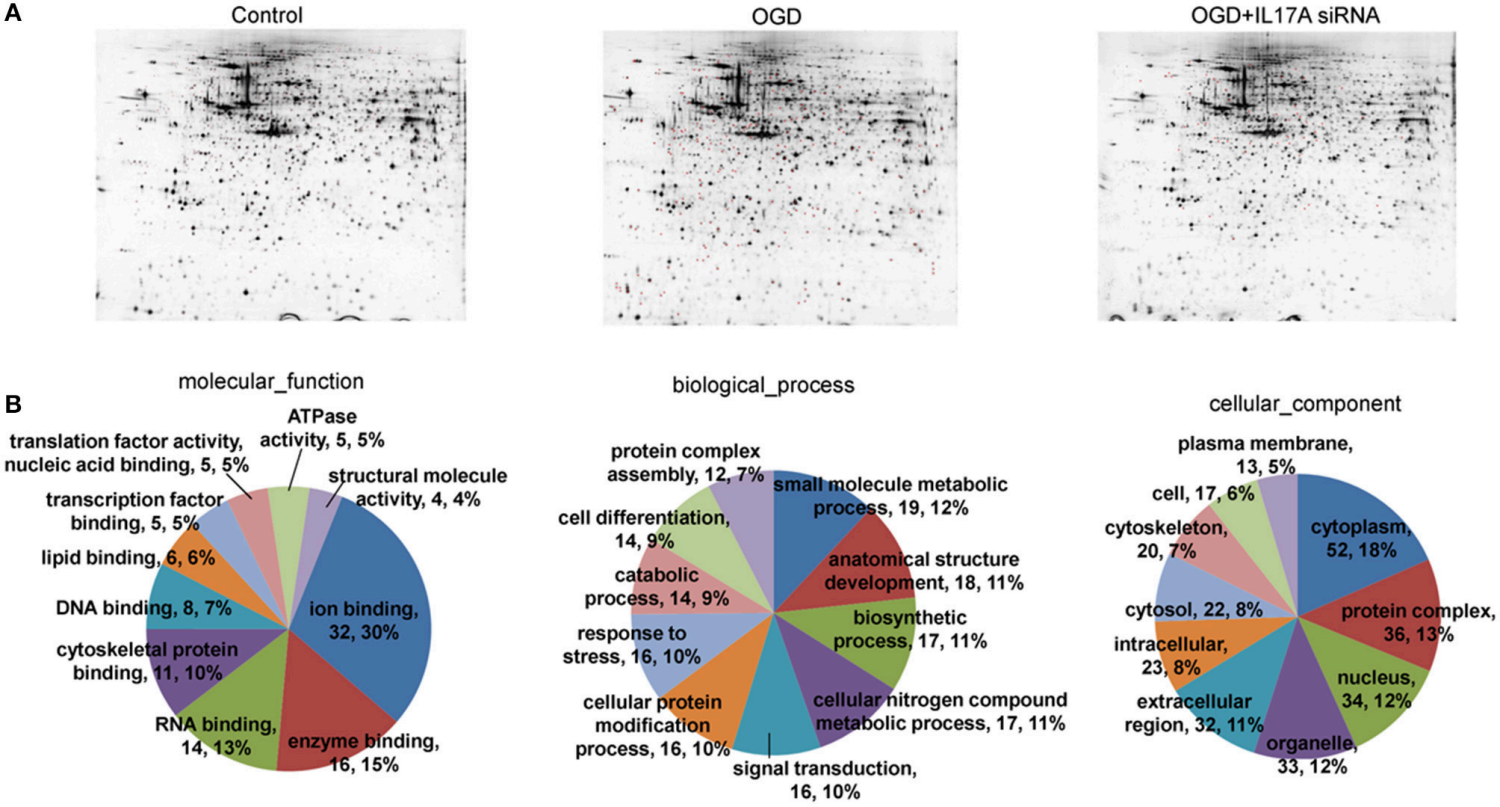
2D-gel electrophoresis and GO analysis. **(A)** Two-dimensional gel electrophoresis (2-DE) of EOC 2 cells (Control), EOC 2 cells in an OGD model (OGD), and EOC 2 cells transfected with IL-17A siRNA in an OGD model (OGD+IL-17A siRNA). Selected protein spots were numbered and collected for identification by MALDI-MS. **(B)** GO molecular function classification of proteins whose expression was changed in the OGD group but elevated in the OGD + IL-17A siRNA group.

To investigate the mechanism of IL-17A in microglial cells, we used mass spectrometry to identify the protein expression profile regulated by IL-17A. Microglial cells were assigned into three groups, including the control group, the OGD group and the OGD + IL-17A siRNA group. Total protein was isolated from the microglial cells in the different groups, and the protein was separated using 2D gel electrophoresis. Then, each SDS-PAGE gel was scanned using a UMAX image scanner. As shown in Figure 4A, the altered expression of some proteins was seen in the OGD and OGD + IL-17A siRNA group. In addition, the proteomic profiles captured from 9 gels were analyzed using imaging analysis software. In the results of Mass spectrometric analysis, the gel points altered in OGD group and recovered in OGD + IL-17A siRNA group were selected for the subsequent identification. The proteins extracted from the spots with an average two-fold or greater changes (*P* < 0.05) were identified with MALDI-TOF/TOF-MS. After an in-gel tryptic digestion, MS analysis, an online identification of the peptide MS database using Mascot, and blasting the NCBI protein sequence database, the differentially expressed proteins were finally identified according to their Mascot scores, the molecular weight shown on the gel, and the isoelectric point information. As shown in Table 1 and Table S1, according to their function, these differentially expressed proteins could be classified into categories, such as signal transduction, cell proliferation, apoptosis, and oxidative stress-related proteins, as well as proteins with unknown functions.

**Table 1:** Differentially expressed proteins in microglial cells in the model of OGD^a^.

**Protein name**	**Accession^b^**	**MW**	**pI**	**Score^c^**	**Coverage (%)**	**No. peptides**	**Pep match^d^**
**DOWN-REGULATED PROTEINS**							
Dihydropyrimidinase-related protein 2	DPYL2_MOUSE	62,638	5.95	661	43	19	107
Heterogeneous nuclear ribonucleoprotein K	B2M1R6_MOUSE	48,760	5.38	466	32	15	77
RuvB-like 2	RUVB2_MOUSE	51,252	5.49	671	47	22	110
NSFL1 cofactor p47	NSF1C_MOUSE	40,655	5.04	626	54	17	140
Serine/threonine-protein phosphatase	Q8BN07_MOUSE	33,136	5.37	476	58	15	89
Thioredoxin-like protein 1	TXNL1_MOUSE	32,616	4.84	622	47	12	150
Eukaryotic translation initiation factor 6	IF6_MOUSE	27,007	4.63	482	52	8	118
Elongin-B	ELOB_MOUSE	13,219	4.87	225	77	8	90
Calponin	A0A0G2JDV8_MOUSE	31,546	5.79	216	37	11	64
Twinfilin-1	TWF1_MOUSE	40,283	6.21	292	26	9	91
**UP-REGULATED PROTEINS**							
Vimentin	VIME_MOUSE	53,712	5.06	230	39	13	54
Actin, cytoplasmic 2	ACTG_MOUSE	42,108	5.31	454	41	11	123
Ubiquitin thioesterase OTUB1	OTUB1_MOUSE	31,478	4.85	567	65	14	138
Phosphoserine phosphatase	SERB_MOUSE	25,308	5.81	486	58	12	95
Caspase-3	CASP3_RAT	31,927	5.92	280	30	7	105
High mobility group protein B1	HMGB1_RAT	25,049	5.62	205	29	6	84
Serine/threonine-protein phosphatase 2A catalytic subunit beta isoform	PP2AB_MOUSE	36,123	5.21	471	51	14	77
BAG family molecular chaperone regulator 2	BAG2_MOUSE	23,630	6.01	292	33	6	68
Annexin	B0V2N8_MOUSE	19,698	5.68	340	43	5	98
Neudesin	NENF_MOUSE	18,893	5.13	271	54	5	67

a *Microglial cells were placed in an oxygen deprived (95% N_2_/3% CO_2_/2% O_2_) incubator at 37 °C for 2 h, and then returned to normoxic conditions with regular medium to terminate the OGD and start reperfusion for 48 h and subjected to 2D gel analyses*.

b *Accession numbers of proteins were derived from Uniprot database*.

c *Probability scores are based on the MS/MS analysis*.

d *Number of peptides matched*.

### Bioinformatics classification analysis and validation of the differentially expressed proteins

**Figure 5:**
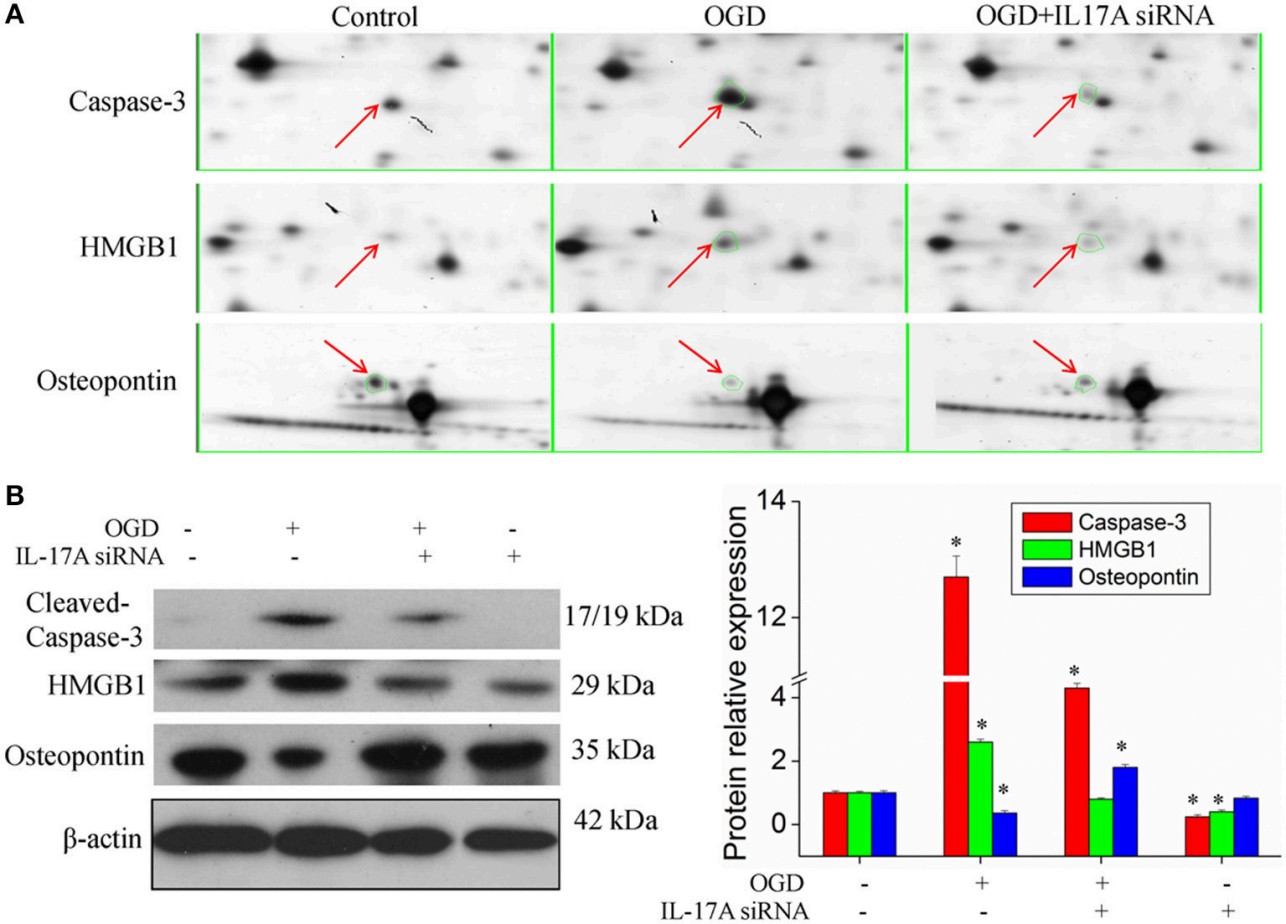
Validation of IL-17A regulatory proteins. **(A)** Typical examples of some differentially expressed protein spots with at least two-fold changes in EOC 2 cells, EOC 2 cells in an OGD model and EOC 2 cells transfected with IL-17A siRNA in OGD model by 2-DE. **(B)** EOC 2 cells were transfected with IL-17A siRNA for 24 h, then started the OGD for 2 h and reperfusion for 24 h, and the expression of cleaved-caspase-3, HMGB1 and osteopontin was measured by Western blot analysis. *P < 0.05 vs. the control.

Figure 4B, Tables S2, S3 show the GO classification (including CC, MF, BP) of the MS-identified proteins. These differential proteins are involved in processes, such as oxidative stress, cell metabolism, biological regulation, etc. For signal transduction pathways, part of the proteins are mainly involve in the apoptosis, p53 signaling pathway, the PI3K/Akt signaling pathway, the Toll-like receptor signaling pathway, etc. (Tables S4, S5). Caspase-3 is a key protease of apoptosis. HMGB1 plays a significant role in cerebral IRI. Osteopontin (OPN) is a key factor in the central nervous system repair and extracellular matrix remodeling after injury. Thus, the proteins Caspase-3, HMGB1, and Osteopontin were selected for validation. The results of the Western blot analysis showed that the protein expression of Caspase-3, HMGB1, and Osteopontin in the EOC 2 cells were consistent with the results of the MS (Figure 5). Therefore, in the microglial cells, IL-17A alters the expression of proteins related to signaling transduction, cell cycle, apoptosis, inflammation, and thereby regulates the activities of many signaling pathways to exert its anti-proliferation and pro-apoptotic effects.

### IL-17A enhances microglia damage in an OGD model through regulation of the p53 and PI3K/Akt pathways

**Figure 6:**
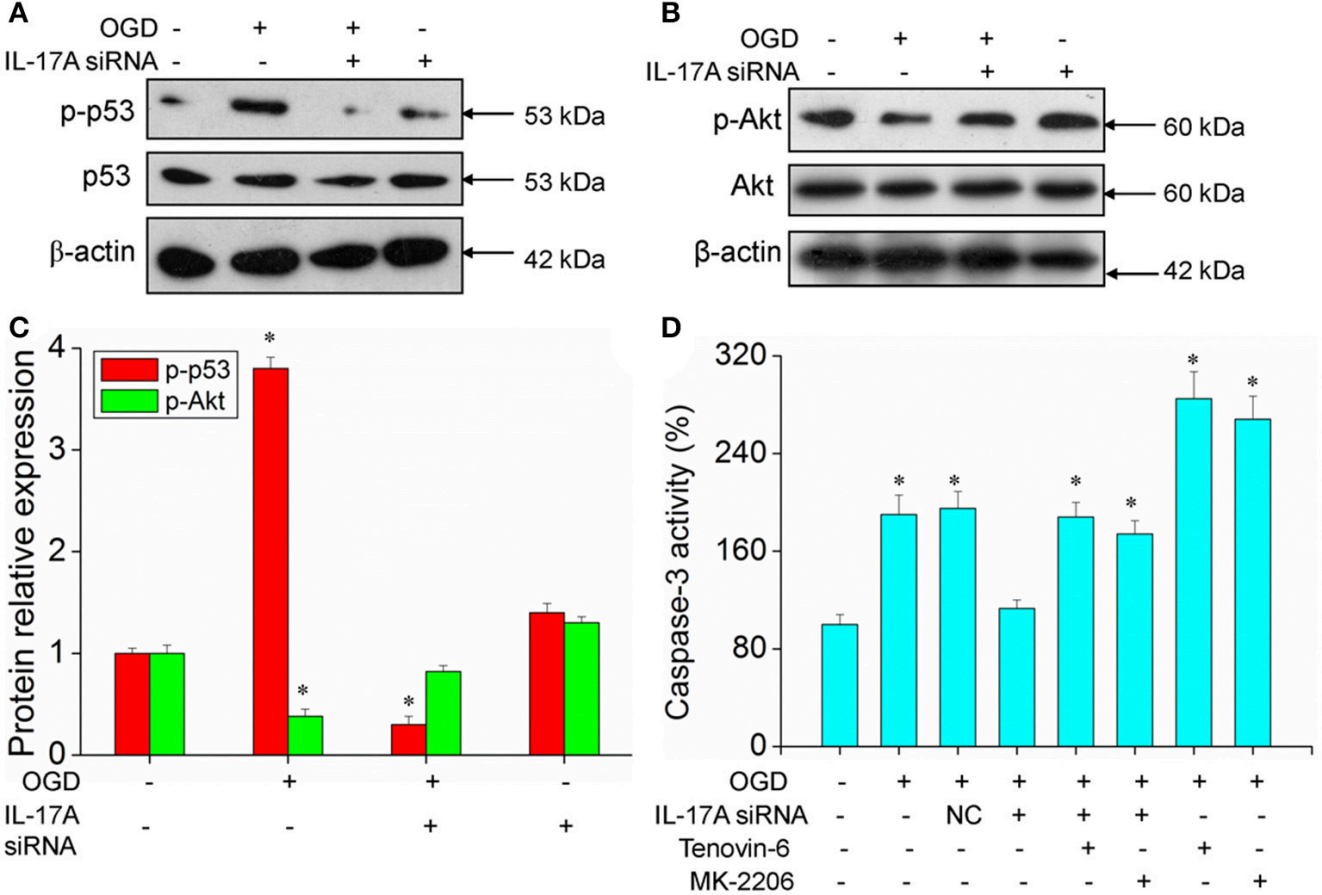
Expression of p-p53 and p-Akt, caspase 3 activity in microglial cells. After transfection with IL-17A siRNA for 24 h, EOC 2 cells were started in the OGD condition for 2 h and reperfusion for 24 h. P-p53 **(A)** and p-Akt **(B)** expression as measured by Western blot analysis. **(C)** Data are presented as the means ± *SD* from three independent experiments, *P < 0.05 vs. the control. **(D)** After EOC 2 cells reperfusion for 2 h, Tenovin-6 (10 μM) or MK-2206 (5 μM) were added or not, and the activity of caspase-3 was monitored by fluorometric method. *P < 0.05 vs. the control.

**Figure 7:**
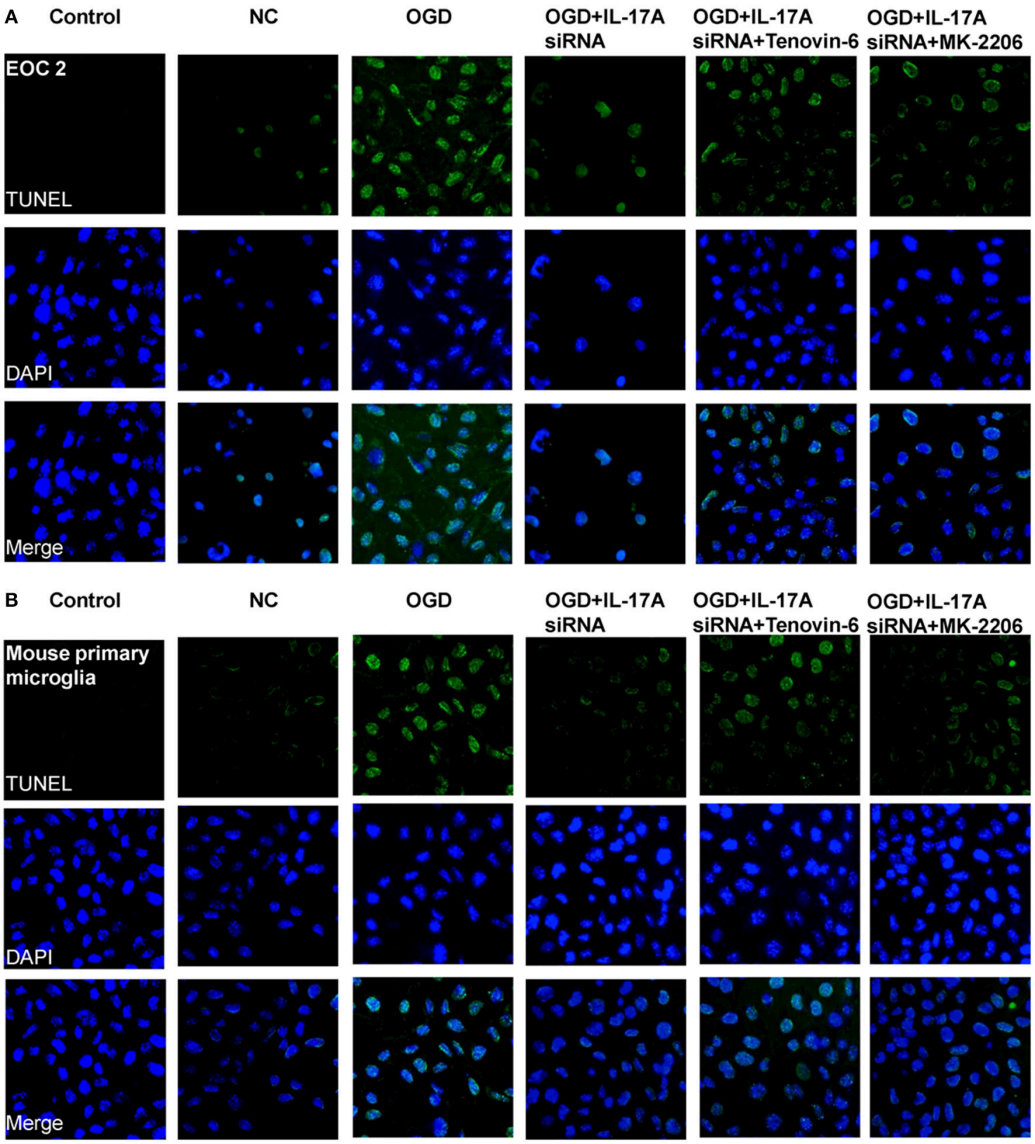
IL-17A enhances microglia apoptosis in OGD model through regulation of p53 and PI3K/Akt pathways. After transfection with IL-17A siRNA for 24 h, EOC 2 cells **(A)** and mouse primary microglia **(B)** were started in the OGD condition for 2 h and reperfusion for 24 h. Tenovin-6 (10 μM) or MK-2206 (5 μM) were added or not after microglial cells reperfusion for 2 h. Then, apoptosis was examined by a TUNEL-DAPI co-staining assay. Green fluorescence indicates the DNA fragmentation, and blue fluorescence indicates the cell nucleus.

Some important downstream pathways were selected for validation, including the p53 and PI3K/Akt signaling pathways. An increase was observed in the phosphorylation level of p53 in the OGD model, while the down-regulation of IL-17A expression caused a significant reduction in the phosphorylation level of p53 (Figures 6A,C). In addition, the phosphorylation level of Akt significantly decreased in the OGD model, while the down-regulation of IL-17A expression resulted in a restoration of the phosphorylation level of Akt (Figures 6B,C). In the OGD model, when an IL-17A siRNA transfection, Tenovin-6 (p53 activator), or MK-2206 (Akt inhibitor) was added, and then, the caspase-3 activity and apoptosis were detected. The results showed that Tenovin-6 or MK-2206 reversed the inhibitory effect of IL-17A siRNA on the caspase-3 activity (Figure 6D) and increased the apoptosis of EOC 2 cells and mouse primary microglia (Figure 7). This indicates that IL-17A regulated the occurrence of injury in the OGD model through the p53 and PI3K/Akt pathways. These findings demonstrate the role of IL-17A in cerebral IRI by affecting a series of important signaling transduction pathways and the expression of some important factors.

### IL-17A and HMGB1 are regulated mutually to exhibit a synergistic effect

**Figure 8:**
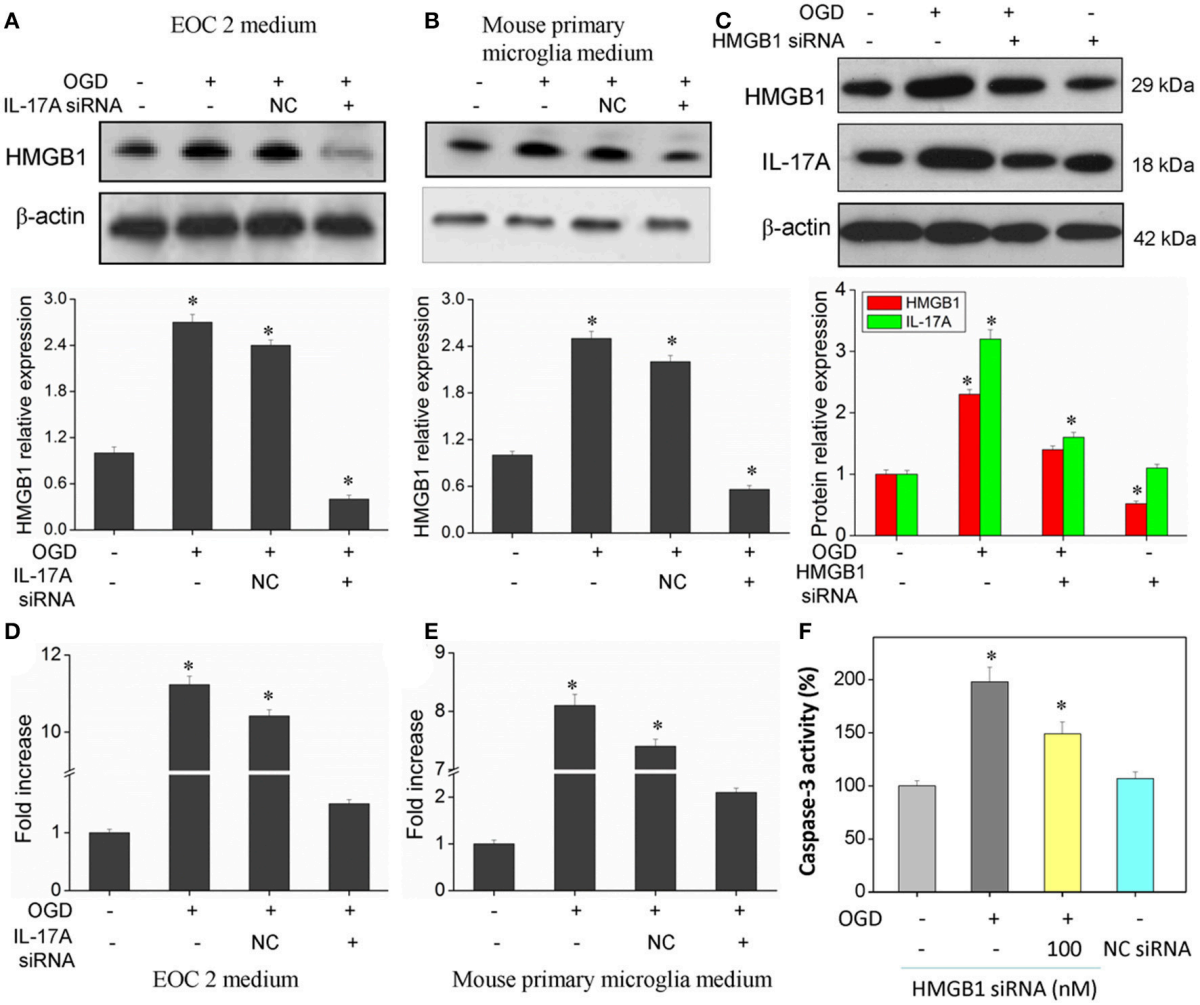
Expression of HMGB1 and IL-17A, and caspase 3 activity in microglial cells. After transfection with IL-17A or HMGB1 siRNA for 24 h, microglial cells were started in the OGD condition for 2 h and reperfusion for 24 h. HMGB1 and/or IL-17A expression as measured by Western blot analysis in EOC 2 culture supernatants **(A)**, mouse primary microglia culture supernatants **(B)**, and EOC 2 cells **(C)**. Protein concentrations of HMGB1 in EOC 2 **(D)** and mouse primary microglia **(E)** culture supernatants were determined by ELISA. **(F)** The activity of caspase-3 was monitored by the fluorometric method. *P < 0.05 vs. the control.

Of the factors that IL-17A regulates, HMGB1 is reported to promote the development of cerebral IRI (Kim et al., 2008; Yang et al., 2010; Zhang et al., 2014). In addition, HMGB1 promotes IL-17A expression in T cells (He et al., 2012; Shi et al., 2012; Zhu et al., 2013). It is therefore hypothesized that HMGB1 and IL-17A may be regulated mutually during cerebral IRI, thereby aggravating the injury. Our findings showed an elevated expression of both HMGB1 and IL-17A in the model of OGD (Figure 8), and the down-regulation of HMGB1 expression caused a reduced IL-17A expression (Figure 8C), while the down-regulation of IL-17A expression resulted in a reduction of HMGB1 expression (Figures 8A,B,D,E). The results demonstrate a synergistic effect between HMGB1 and IL-17A through mutual regulation. We further assessed the effect of HMGB1 on Caspase-3 activity, and our findings showed that down-regulation of HMGB1 expression reduced Caspase-3 activity in the OGD model, indicating that HMGB1 has a pro-apoptotic action (Figure 8F).

### Relationship between ROS, IL-17A, HMGB1, and p53, PI3K/Akt pathways

**Figure 9:**
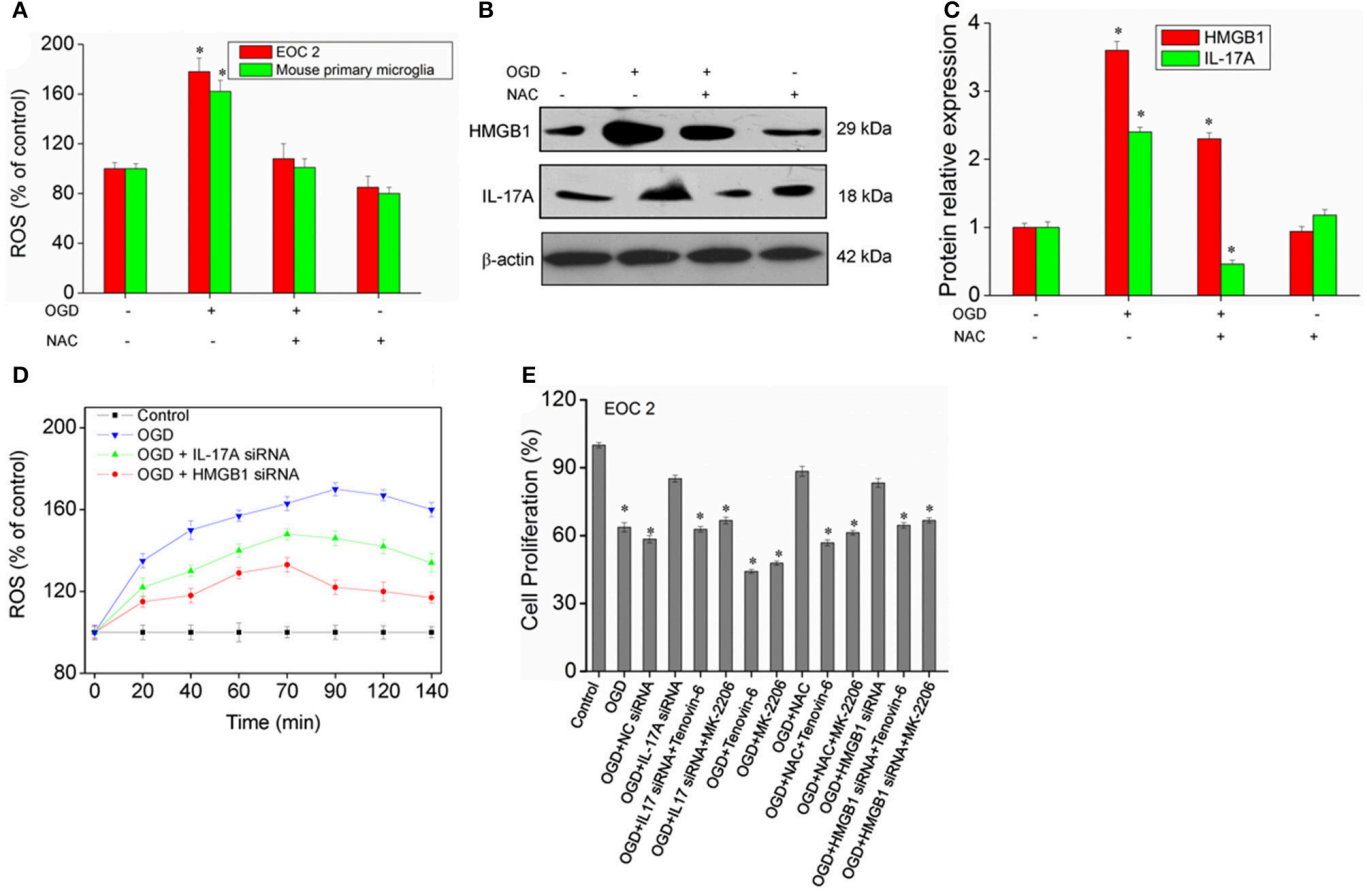
ROS production and expression of HMGB1, IL-17A in microglial cells. **(A)** Overproduction of ROS in microglial cells exposed to OGD/reperfusion or/and NAC. **(B,C)** After treatment with NAC, EOC 2 cells were started in the OGD condition for 2 h and reperfusion for 24 h, then HMGB1 and IL-17A expression as measured by Western blot analysis. *P < 0.05 vs. the control. **(D)** After transfection with IL-17A or HMGB1 siRNA for 24 h, EOC 2 cells were started in the OGD for 2 h and reperfusion for 24 h, and ROS production was then examined for an indicated time. **(E)** After transfection with IL-17A or HMGB1 siRNA for 24 h, EOC 2 cells were started in the OGD condition for 2 h and reperfusion for 24 h. NAC and/or Tenovin-6 (10 μM) or MK-2206 (5 μM) were added or not after microglial cells reperfusion for 2 h. Then, cell proliferation was examined by BrdU assay. *P < 0.05 vs. the control.

A large amount of ROS is produced during cerebral ischemia and reperfusion (Kahles and Brandes, 2012), and mass spectrometry demonstrates that IL-17A correlates with oxidative stress. It is therefore speculated that there may be a relationship between ROS and IL-17A. In the model of OGD, the amount of ROS was found to almost double in the microglial cells, and the production of ROS almost recovered to the level in the control group following the addition of the ROS inhibitor N-acetyl-L-cysteine (NAC) (Figure 9A). In addition, an obvious down-regulation was seen in the expression of IL-17A and HMGB1, and notably, IL-17A and the up-regulation of IL-17A expression induced by OGD/reperfusion were completely blocked (Figures 9B,C). The results demonstrated that the production of a large amount of ROS may promote IL-17A and HMGB1 expression, and the down-regulation of IL-17A or HMGB1 expression caused a reduction in the production of ROS (Figure 9D), which, however, still appeared as an increasing trend relative to the control group. It is therefore considered that ROS may be located upstream of HMGB1 and IL-17A. Furthermore, IL-17A siRNA, NAC, and HMGB1 siRNA increased the microglial cell proliferation in the *in vitro* model of OGD, but p53 activator Tenovin-6 or Akt inhibitor MK-2206 blocked the above effects (Figure 9E). So, ROS, IL-17A, and HMGB1 all can regulate the microglial cell proliferation through p53 and PI3K/Akt pathways.

## Discussion

Cerebral IRI is a pathophysiological phenomenon of severe brain cell damage induced by the restoration of blood flow perfusion after cerebral ischemia (Chen et al., 2013). Currently, the role of cytokines in IRI has received much attention, and IL-17, which is secreted by Th17 cells, is demonstrated to play an important role in IRI (Weaver et al., 2006; Shichita et al., 2009).

In this study, by targeting microglial cells, a model of OGD was established to simulate the development of cerebral IRI *in vitro*, and IL-17A expression was significantly up-regulated in the OGD group. In addition, the proliferation of microglial cells was inhibited, while apoptosis increased in the OGD group. In addition, the down-regulation of IL-17A expression caused a increase in microglial cell proliferation and a reduction in the apoptosis of microglial cells. The findings demonstrate that IL-17A promotes the apoptosis in microglial cells and inhibits cell proliferation. The caspase family is a class of proteases that plays a critical role in apoptosis. In this present study, the activities of Caspase-3, Caspase-8, Caspase-9, and PARP were all remarkably elevated in the microglial cells in the OGD model, while the down-regulation of IL-17A expression resulted in a significant reduction in the activity of Caspase-3, Caspase-8, Caspase-9, and PARP, demonstrating that IL-17A has a pro-apoptotic role. Liao et al. (2012) found that IL-17A, mainly produced by γδT cells, played a pathogenic role in myocardial I/R injury by inducing cardiomyocyte apoptosis and neutrophil infiltration.

To date, there is little knowledge on the mechanism underlying the role of IL-17A in cerebral IRI. Mass spectrometric identification revealed that IL-17A affected the expression of a series of proteins in the OGD model, and most of these proteins were involved in oxidative stress, cell metabolism, apoptosis, and signal transduction through the p53, PI3K-Akt, and TLR signaling pathways. The expression of Caspase-3, HMGB1, and Osteopontin was validated using a Western blotting analysis and was in agreement with the mass spectrometric results. It is therefore considered that IL-17A affects the p53 and PI3K-Akt signaling pathways by regulating the expression of factors associated with oxidative stress and apoptosis to act in cerebral IRI.

In the OGD model, our findings showed a significant increase in the p53 phosphorylation level and a decrease in the Akt phosphorylation level, and the down-regulation of IL-17A expression caused a significant decrease in the p53 phosphorylation level and restoration of the Akt phosphorylation level. Additionally, the results also showed that Tenovin-6 (p53 activator) or MK-2206 (Akt inhibitor) reversed the inhibitory effect of the IL-17A siRNA on the caspase-3 activity, and increased the apoptosis of the EOC 2 cells and the mouse primary microglia. The results demonstrated that the p53 expression induced by cerebral IRI was consistent with the emergence of apoptosis (Engelhard et al., 2004). The inhibition of the p53 gene expression suppresses the development of nerve cell apoptosis, which protects ischemic brain injury (Panahian et al., 1999). As an important signaling pathway involved in the regulation of cell proliferation, the PI3K/Akt signaling transduction pathway is a key signaling molecule in multiple vital activities. Akt, which is central to this signaling pathway, is not only the direct target downstream of PI3K, it is also the primary target enzyme of PI3K. The level of phosphorylated Akt showed a slow and persistent reduction in ischemic brain tissues, while hypoxic preconditioning restored the level of phosphorylated Akt and its substrate glycogen synthase kinase 3 beta (GSK3B) reduced the release of the pro-inflammatory mediators NF-kB, COX-2, and CD68, thereby alleviating necrosis of the ischemic cerebral tissues (Yin et al., 2007). The results of these studies demonstrate that IL-17A functions in cerebral IRI by affecting a series of important signal transduction pathways and the expression of some important factors.

Of the factors medicated by IL-17A, the role of HMGB1 is clearly illustrated. Recently, HMGB1 was found to correlate with IRI (Kim et al., 2008; Yang et al., 2010; Zhang et al., 2014). Following acute ischemic brain injury, the release of a large amount of HMGB1 induces an inflammatory reaction, such as the activation of microglial cells (Qiu et al., 2008). In addition, HMGB1 is reported to obviously induce the activation of microglial cells, astrocytes and microvessels 2 days post-reperfusion, and this is sustained for several days (Kim et al., 2008). It is therefore hypothesized that IL-17A promotes the development of injury by up-regulating HMGB1 expression. However, it is reported that HMGB1 promotes IL-17A expression (Zhu et al., 2013; Zhang et al., 2014). Our findings showed that the expressions of HMGB1 and IL-17A siRNA were affected mutually, indicating the mutual interplay between HMGB1 and IL-17A siRNA. The results of the current study also demonstrated that the down-regulation of HMGB1 expression reduced Caspase-3 activity in the model of OGD, indicating that HMGB1 and IL-17A are regulated mutually to show a synergistic action to promote the development of microglial cell apoptosis.

IL-17A promotes ROS production (Wang et al., 2009; Pietrowski et al., 2011), Pietrowski et al. (2011) indicated that IL-17A caused the NAD(P)H-oxidase dependent generation of ROS, leading to the proinflammatory activation of vascular smooth muscle cells (VSMC). Moreover, mass spectrometry reveals that IL-17A is associated with oxidative stress. It is indicated that a large amount of ROS is produced during cerebral ischemia and reperfusion (Kahles and Brandes, 2012). It is therefore assumed that there is a correlation between ROS and IL-17A. Our findings showed that the addition of the ROS inhibitor NAC effectively inhibited the up-regulation of HMGB1 and IL-17A expression induced by OGD, and accordingly, HMGB1 and IL-17A siRNA affected ROS at different degrees, indicating that HMGB1 and IL-17A affect ROS production through a feedback mechanism. However, the down-regulation of IL-17A or HMGB1 expression did not completely block the production of ROS, suggesting that ROS is located upstream of HMGB1 and IL-17A. Kotla et al. (2013) also found that ROS production led to the Syk-, Pyk2-, and mitogen-activated protein kinase (MAPK)–dependent production of the proinflammatory cytokine IL-17A in a manner that required the transcription factor CREB (cyclic adenosine monophosphate response element-binding protein).

Further, ROS, IL-17A, and HMGB1 all can regulate the microglial cell proliferation through p53 and PI3K/Akt pathways. Ge et al. (2016) indicated that NAC inhibited p53 expression, and decreased the apoptosis of HAPI microglia. Chen et al. (2016) found that hypoxia/reperfusion can evoke autophagy-activated microglia apoptosis/death via an ROS-regulated Akt/mTOR signaling pathway, which can be reversed by catechin. Zhou et al. (2015) also suggested that the ROS-dependent increase in phosphatase and tensin homolog activity in reperfusion period relieves ERK1/2 from inhibition of Akt. And Hu et al. (2011) proved that PI3K/Akt signaling pathway involved in cardioprotection of preconditioning with HMGB1 protein during myocardial ischemia and reperfusion. Above results confirmed that ROS and HMGB1 can regulate the p53 and PI3K/Akt pathways. Min et al. (2017) found that hypoxia induced translocation of HMGB1 into the extracellular area and it was dependent on ROS produced by dual oxidase 2. This further proved that ROS can regulate the functions of HMGB1.

OGD/R significantly decreased the cell viability and increased the release of IL-1β, IL-6, IL-8, IL-10, TNF-α in BV2 microglia cells (Zhou et al., 2016). IL-17A siRNA increased the microglial cell proliferation in the *in vitro* model of OGD. These suggested that the decreasing proliferation and enhanced apoptosis maybe a harmful factor. Lu et al. (2017) found that Oxysophocarpine reduced OGD/R-induced inflammation in BV-2 microglia and suppressed OGD/R-elicited BV-2 cell apoptosis. Ye et al. (2017) show that CHPG (the selective mGluR5 agonist) pretreatment, protected BV2 cells against OGD/R-induced cytotoxicity, apoptosis, the release of inflammatory cytokines, and the accumulation of ROS. In a study of Alzheimer's disease, the inhibition of p53 led to a decrease in microglial apoptosis and prevented microglial neurotoxicity (Davenport et al., 2010). By contrast, the up-regulation of p53 expression in ischemic brains increased microglial apoptosis and BBB damage, and worsened brain damage (Tu et al., 2012). Preventing microglia death may, therefore, provide a strategy for promoting tissue restoration after stroke. Thus, ROS, IL-17A, and HMGB1 were found to play a critical role in cerebral IRI. In summary, a large amount of ROS is produced during cerebral ischemia and reperfusion, thereby promoting HMGB1 and IL-17A expression, and the up-regulation of IL-17A expression mediates the expression of a series of factors, which affect the p53 and PI3K/Akt signaling pathways and thereby promote apoptosis and aggravate injury. Lv et al. (2016) indicated that Sphk1/S1P regulates the expression of IL-17A in activated microglia, inducing neuronal apoptosis in cerebral ischemia/reperfusion. In the *in vitro* model of OGD, IL-17 showed a dose-dependent effect in promoting neuronal injury through IL-17-IL-17R combination which can be blocked by IL-17R/Fc chimera (Wang et al., 2009).

Such a finding would provide exact evidence for clarifying the pathogenesis of cerebral IRI and provide new insights into the alleviation of cerebral IRI. But unfortunately, this study was carried out only in microglia. More research needs to be done at other cells or in animals.

## Author contributions

BZ and NY designed and conducted most of the experiments. ZM, SL, and FZ designed and conducted some of the experiments. BZ and NY wrote most of the manuscript. All the authors analyzed the data, revised the manuscript, and approved the final manuscript.

### Conflict of interest statement

The authors declare that the research was conducted in the absence of any commercial or financial relationships that could be construed as a potential conflict of interest.
