# Supplementary material for: IL-17A Enhances Microglial Response to OGD by Regulating p53 and PI3K/Akt Pathways with Involvement of ROS/HMGB1
Source: Front Mol Neurosci. 2017 Aug 31;10:271. doi: 10.3389/fnmol.2017.00271 (PMC5583146; doi:10.3389/fnmol.2017.00271)
Supplement: Supplementary file 6 [file DataSheet1.DOC]

**IL-17A enhances microglial response to OGD by regulating p53 and PI3K/Akt pathways with involvement of ROS/HMGB1**

Bin Zhang^1†^, Ning Yang^1†*^, Zhi-ming Mo^1^, Shao-peng Lin^2^, Feng Zhang^3^

**Protein extraction**

The collected cells were added with l mL protein lysis buffer (7 M urea, 2 M thiourea, 4% CHAPS, 30 mM Tris, and protease inhibitors; pH 8.5), sonicated on ice bath, and centrifuged at 4 °C, 12,000 r/min, for 20 min. The supernatants were collected, added with 4 volumes of acetone, and precipitated overnight at -20 °C. After precipitation, the proteins were centrifuged at 4 °C, 12,000 r/min, for 20 min. The supernatants were removed, and the tubes were naturally dried with open lid on a clean paper towel. The obtained protein pellets were stored at -80 °C until further use.

**The 2D-gel electrophoresis**

The protein samples in each group were mixed with 450 μL rehydration solution (8 M urea, 2% CHAPS, 13 mM DTT, 0.5% IPG buffer, 0.002% bromophenol blue) respectively. The amount of protein sample loaded on each gel was 300 μg, and 3 gel repeats were performed for each group, to make ​​the 1st dimension isoelectric focusing system, and the samples were distributed evenly. The parameters for isoelectric focusing were as follows: 30 V, 12 h; 500 V, 1 h; 1000 V, 1 h; and 8000 V, 8 h. After isoelectric focusing, the gel strips were equilibrated in equilibrium solution I (1% DTT, 50 mM Tris-HCl, 6 M urea, 30% glycerol, 2% SDS, and 0.002% bromophenol blue) and equilibrium solution II (4% iodoacetamide, 50 mM Tris-HCl, 6 M urea, 30% glycerol, 2% SDS, and 0.002% bromophenol blue) respectively for 15 min each time, transferred to the prepared 2nd dimension 12.5% SDS-PAGE, and overlaid with 0.5% agarose gel, to perform the 2nd dimension electrophoresis. The electrophoresis parameters were as the following: 30 W, 45 min and 90 W, 6 h. The electrophoresis was terminated when the bromophenol blue dye migrated to the edge of the gel.

**Image analysis and protein identification**

The silver-stained 2D-gel was scanned into computer with imagescanner, and each gel image was analyzed using ImageMaster^TM^ 2D Platinum 7.0 software. The images of control group (3 gels) were matched with the images of treatment group (3 gels); the protein spot parameters were set as Smooth=10, Saliency=2, and Min Area=5, to search for the identical and differential protein spots between different groups, and calculate the relative amount of protein on the same spot. Meanwhile, Student's t-test and Mann-Whitney U test were used to analyze statistical differences (*P*<0.05). Since there were 3 repeats in each group, there were 3 paralleled 2D-gels, and the one with gel image showing relatively clear protein spots was selected for subsequent MS analysis.

**Tryptic digestion and MALDI-TOF mass spectrometry**

The selected protein spots were cut out precisely, placed into respective 0.25 mL silanized centrifuge tubes, added with 100 μL 0.1 mM NH_4_HCO_3_/50% acetonitrile, and shaken twice at room temperature for 45 min each time, until the gel piece became colorless. After removing the supernatant, the gel was freeze-dried, added with 10 mM DTT, 40 mM NH_4_HCO_3_ solution, incubated at 56 °C for 1 h. The supernatant was then removed, and the gel was added with equal volume of 55 mM iodoacetamide, 40 mM NH_4_HCO_3_ solution, and continuously shaken in the dark for 45 min. After removing the supernatant, the gel was freeze-dried, added with 5 μL of trypsin (20 μg/mL) and 5 μL 40 mM NH_4_HCO_3_ solution, smashed, and incubated at 4 °C for 30 min. The reaction was then added with 10 μL 40 mM NH_4_HCO_3_ solution. After incubating at 37 °C for another 16 h, the supernatant was transferred to a new centrifuge tube. The original tube was added with 100 μL 50% acetonitrile, 0.1% trifluoroacetic acid (TFA), and sonicated on ice bath for 5 min. The 2 batches of supernatant were combined, freeze-dried, and dissolved in 5 μL 0.1% TFA. After desalting through ZipTip C18, 0.5 μL sample was mixed with 0.5 μL CCA and analyzed on ​​MALDI-TOF/TOF. The peptide mass fingerprints obtained from MS were used to search for matching protein against the NCBI database, according to the type of protein source, number of matched peptide fragments, the protein score, the confidence interval (*CI%*) of the protein score, etc. In general, Score >60 and *CI%* >95 are considered the threshold for successful identification.
